# Male infertility after endoscopic Totally Extraperitoneal (Tep) hernia repair (Main): rationale and design of a prospective observational cohort study

**DOI:** 10.1186/1471-2482-12-7

**Published:** 2012-05-21

**Authors:** Nelleke Schouten, Thijs van Dalen, Niels Smakman, Sjoerd G Elias, Cees van de Water, Roan J Spermon, Laurens Sibinga Mulder, Ine P J Burgmans

**Affiliations:** 1 Department of Surgery/Hernia Clinic, Diakonessenhuis Utrecht/Zeist, Utrecht, The Netherlands; 2 Julius Center for Health Sciences and Primary Care, Utrecht, The Netherlands; 3 Department of Laboratory, Diakonessenhuis Utrecht/Zeist, Utrecht, The Netherlands; 4 Department of Urology, Diakonessenhuis Utrecht/Zeist, Utrecht, The Netherlands; 5 Department of Radiology, Diakonessenhuis Utrecht/Zeist, Utrecht, The Netherlands; 6 Department of Surgery, Diakonessenhuis, Professor Lorentzlaan 76, 3707, HL, Zeist, The Netherlands

## Abstract

**Background:**

To describe the rationale and design of an observational cohort study analyzing the effects of endoscopic Totally Extraperitoneal (TEP) hernia repair on male fertility (MAIN study).

**Methods and design:**

The MAIN study is an observational cohort study designed to assess fertility after endoscopic TEP hernia repair. The setting is a high-volume single center hospital, specialized in TEP hernia repair. Male patients of 18-60 years of age, with primary, reducible, bilateral inguinal hernias and no contraindications for endoscopic TEP repair are eligible for inclusion in this study. Patients with an ASA-classification ≥ III and patients with recurrent and/or scrotal hernias and/or a medical history of pelvic surgery and/or radiotherapy, known fertility problems, diabetes and/or other diseases associated with a risk of fertility problems, will be excluded. The primary outcome is the testicular perfusion before and 6 months after TEP hernia repair (assessed by means of a scrotal ultrasonography). Secondary endpoints are the testicular volume (Ultrasound), semen quality and quantity and the endocrinological status, based on serum levels of the sexual hormones follicle-stimulating hormone (FSH), luteinizing hormone (LSH), testosterone and inhibin B before and 6 months after TEP hernia repair.

**Discussion:**

The use of polypropylene mesh is associated with a strong foreign body reaction which could play a role in chronic groin pain development. Since the mesh in (endoscopic) inguinal hernia repair is placed in close contact to the vas deferens and spermatic vessels, the mesh-induced inflammatory reaction could lead to a dysfunction of these structures. Relevant large and prospective clinical studies on the problem are limited. This study will provide a complete assessment of fertility in male patients who undergo simultaneous bilateral endoscopic TEP hernia repair, by analyzing testicular perfusion and volume, semen quantity and quality and endocrinological status before and 6 months after TEP repair.

**Trial registration:**

The MAIN study is registered in the Dutch Trial Register (NTR2208)

## Background

Inguinal hernia repair is one of the most common interventions in general surgery in the Western world. Worldwide over 20 million inguinal hernia repairs are performed annually [1]. The treatment of an inguinal hernia has changed considerably over the past 15 years. Tension-free repair has become the standard surgical technique in inguinal hernia surgery and has led to a considerable reduction in the recurrence rate [2]. Endoscopic Totally Extraperitoneal (TEP) repair is a safe and cost-effective tension-free technique, if expertise is available [3]. Advantages of this method are its low recurrence rate, fast postoperative recovery, low incidence of chronic postoperative pain and high level of patient satisfaction [4-6].

The use of alloplastic material is a complicated issue. The biomaterial most commonly used in hernia repair is polypropylene, which has good mechanical stability and induces an acute inflammatory reaction followed by a chronic foreign body fibroblastic reaction essential for optimal fixation and incorporation of the biomaterial in the abdominal wall [7]. However, the foreign body reaction could also – together with other factors- play a role in chronic pain development. Moreover, as the mesh is placed in close contact with the vas deferens and the spermatic vessels during (endoscopic, preperitoneal) TEP repair, the changes following the mesh-induced fibrotic reaction could lead to a dysfunction of these structures, which might result in fertility problems [8,9].

In 2005, Shin et al. reported on 14 patients with postoperative obstructive azoospermia after hernia repair with implantation of polypropylene meshes [8]. Their report was soon followed by other case reports and studies focusing on this specific problem [10-12]. Patients considered to be at the greatest risk are fertile men (18-60 years of age) undergoing bilateral mesh repair for inguinal hernias and those who undergo an unilateral repair with impairment of the contra-lateral testis [8].

However, a closer look at the existing literature reveals that most studies on obstructive azoospermia are based on animal experiments and incidental case reports. Relevant clinical – and especially large and prospective - studies on the subject, are limited; up until now, only two studies addressed the problem of fertility after endoscopic TEP hernia repair [13,14].

The importance of well-designed clinical trials to study the risk of mesh-associated fertility was therefore pointed out in the guidelines of the European Hernia Society [3].

The aim of this manuscript is to describe the rationale and design of a prospective, observational cohort study evaluating fertility 6 months after endoscopic TEP hernia repair in male patients with bilateral hernias.

## Methods and design

### Study design

**Figure 1:**
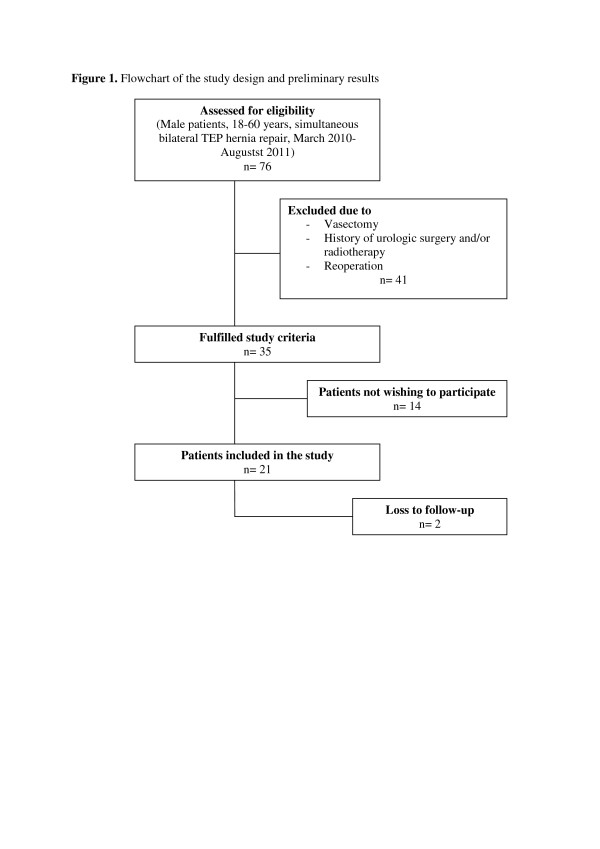
Flowchart of the study design and preliminary results.

The study design is an observational, prospective cohort study involving a high-volume hospital in the Netherlands specialized in the TEP technique for inguinal hernia repair (Diakonessenhuis Utrecht/Zeist). The follow-up of patients is 6 months; parameters of fertility (semen analysis, scrotal ultrasonography and endocrinological status) will be assessed before and 6 months after surgery. A flowchart of the study and the preliminary results (updated until August 15, 2011) is shown in Figure 1. The design, conduct and reporting of this study will adhere to the STROBE guidelines for observational studies [15].

### Patient population

All male patients of fertile age (i.e. 18-60 years old), with primary, bilateral inguinal hernias, who are scheduled for an elective endoscopic Totally ExtraPeritoneal (TEP) hernia repair are eligible for inclusion in this observational study. A total number of 76 patients will be included in the study. Patients will be recruited during their first visit at the outpatient clinic of the participating hospital. Patients are screened for eligibility according to the criteria listed in Table 1.

**Table 1:** Inclusion and exclusion criteria

**Inclusion criteria**	**Exclusion criteria**
**- Male gender**	**- Female gender**
**- Age ≥ 18 and ≤ 60 years**	**- Age ≥ 60 years**
**- Primary, reducible inguinal hernias**	**- Period of high fever prior to semen analysis**
**- Bilateral hernias**	**- Recurrent inguinal hernias**
**- Eligible for TEP (and therefore for general anesthesia)**	**- Unilateral hernias**
	**- Hydrocele and/or varicocele**
	**- Femoral or scrotal hernias**
	**- Incarcerated hernias**
	**- ASA classification ≥ III**
	**- Previous medical history of:**
	**·Testicular infection(s)**
	**·Testicular torsion**
	**·Cryptorchidism**
	**·Inguinal, scrotal, testicular or prostate surgery**
	**·Vasectomy**
	**·Radiotherapy of pelvic region**
	**·Diabetes Mellitus**
	**·Cystic Fibrosis**
	**·Fertility problems and/or treatment, erection disorders or (other) problems in sexual function**
	**·Use of gonadotrofine medication**
	**·Use of anabolic steroids**

### Intake

The study information is sent to all patients with bilateral hernias after an appointment is made for an initial consultation. The appointment will, ultimately within one week, take place with one of the four surgeons and the investigator at the outpatient clinic. After screening for eligibility criteria, informed consent is obtained and the patient is included in the study. Preoperative patient data are obtained by the investigator (Table 2) and are – as standard procedure - recorded in the patient Electronic Patient Chart (Dutch: EPD)

**Table 2:** Preoperative data

**- Age**
**- ASA-classification**
**- Co-morbidity **(see Table 1)
**- Medication**
**- Smoking habit**
**- Body Mass Index (BMI)**
**- Numerical Rating Scale (NRS) to evaluate pain associated with the inguinal hernia**
**- Duration of (hernia associated) symptoms in months**

### Interventions

The perioperative care and surgical technique are not different for patients participating in this trial compared to patients who are not. The applied surgical method is the simultaneous bilateral endoscopic TEP inguinal hernia repair, using a “double-mesh” implantation technique performed under general anesthesia. Two identical polypropylene meshes (Prolene, 12x15 cm) are positioned in a tension-free manner in the preperitoneal space, as described previously [5]. The mesh graft is not fixed, since it reduces operative time, saves costs and avoids possible entrapment neuralgia. Hernia types are classified during TEP repair according to the Nyhus classification. Intra-operative complications and operative time are registered in the Electronic Patient Chart (Dutch: EPD).

Fertility aspects are evaluated by means of scrotal ultrasonography, semen analysis and assessment of the endocrinological status, based on serum levels of the sexual hormones follicle-stimulating hormone (FSH), luteinizing hormone (LSH), testosterone and inhibin B.

A scrotal ultrasonography is performed to investigate bilateral testicular volume and to detect possible signs of distension of the epididymis and/or vas deferens [16].

The flow in the testicular artery is measured by Doppler ultrasonography measuring the blood flow velocity (cm/s) in the testicular, capsular and intratesticular arteries. The parameters that are to be evaluated are described in the Additional file 1 Addendum A. Scrotal ultrasound is performed by one experienced radiologist.

Semen analysis is done in a standardized manner. Classic parameters, such as seminal volume (mL), sperm concentration (10^6^ cells/mL), motility (% progression) and morphology (% normal) are analyzed. Patients are instructed about minimal (2 days) and maximal (7 days) abstinence so that reliable semen samples can be obtained.

Fertility aspects are evaluated preoperatively (on the day of surgery) and 6 months postoperatively.

### Postoperative management and follow-up

Patients are discharged on the day of surgery, unless complications occur. Duration of hospital stay and postoperative complications are registered in the EPD. At discharge, patients are advised to take pain medication (Paracetamol and if necessary Diclofenac) when necessary and avoid strenuous physical activity (lifting, sports) during the first postoperative week. There are no other (physical) restrictions. Patients undergo a (physical) examination 6 weeks and 6 months after surgery. Surgery related complications, the time that it takes to restart normal activities pain status, evaluated by the Numerical Rating Scale (NRS) and the presence of recurrent hernias are evaluated by an experienced surgeon. The 6 monthly visit to the outpatient clinic is combined with the assessment of fertility aspects (see “*Intervention”*).

### End-points

The primary endpoint is the testicular perfusion before and 6 months after endoscopic TEP hernia repair, as measured by Doppler ultrasonography.

Secondary endpoints are the testicular volume, semen quality and quantity and serum levels of sexual hormones (FSH, LH, testosterone and inhibin B), evaluated before and 6 months after endoscopic TEP hernia repair.

### Safety measures

All four surgeons have extensive experience with TEP repair (over 500 procedures per surgeon). Standard safety measures are applied at all times. The operation and perioperative care are not different for patients participating in this study compared to patients who do not participate. Therefore, serious adverse events (SAE), other than SAE’s that might eventually occur following endoscopic TEP repair (hemolytic shock, bladder or bowel laceration) are not expected to occur in this study.

### Sample size and power

Since insufficient human data are available in the current literature regarding fertility aspects (scrotal ultrasound data, semen characteristics) after inguinal hernia repair in men, no adequate power analysis, and consequently no adequate sample size calculation, can be performed.

However, in two studies regarding testicular perfusion after endoscopic TEP hernia repair, a difference was found in the end diastolic velocity (EDV) of the a. testicularis (as a parameter of testicular perfusion). The follow-up was respectively 7 days and 3 months after TEP hernia repair; mean differences (δ) of respectively 0.1 cm/s and 1.1 cm/s were observed in the EDV of the a. testicularis [17,18].

Based on these findings, an assumption is made that, if the testicular perfusion 6 months after TEP repair is compared with the perfusion preoperatively and a δ of 0.4 cm/s is detected in the EDV of the a. testicularis, 76 patients have to be included in this study with a two sided alpha of 0.05 and a power of 0.90. In this calculation, a loss to follow-up of 15% is expected.

### Statistical methods

SPSS software (SPSS, Chicago, Illinois, USA) will be used for statistical analysis. Normality of continuous data will be evaluated with the Shapiro Wilk test. Homogeneity of variances will be checked by means of Levene’s analysis. Differences in preoperative and postoperative fertility parameters will be analyzed by means of a paired sample *T*-test (parametric data) or a Wilcoxon signed-ranked test (non-parametric data). Data will be compared to the age-adjusted reference values in the literature. Testicular volume and perfusion will be evaluated for both testes separately. Significance is set at a level of p ≤ 0.05 (two-sided).

## Discussion

Although animal models show (substantial) effects of hernia surgery on the structures in the spermatic cord, the effect being more pronounced in mesh hernia repair, clinical studies indicate that these potential adverse effects do not seem to have a clinical impact on male fertility in humans with inguinal hernias. Then again, the number of studies as well as the included number of patients are limited, making it difficult to draw firm conclusions regarding the effect of mesh hernia repair on male fertility. (Future) studies are therefore needed to further investigate the clinical relevance of the effects of inguinal hernia repair on male fertility.

The MAIN study is a prospective, observational, cohort study designed to provide a complete assessment of fertility in 76 male patients who undergo endoscopic TEP hernia repair for a bilateral hernia, by analyzing testicular perfusion and volume, semen quantity and quality and endocrinological status before and 6 months after TEP repair.

In our hospital, the TEP technique is the preferred operative technique, since endoscopic hernia repair techniques are associated with significantly less postoperative pain and an earlier return to normal activities compared to conventional (open anterior) hernia repair; TEP is preferred over Transabdominal Preperitoneal (TAPP) hernia repair, since it is less invasive and associated with fewer visceral injuries [3].

The main challenge that we anticipate is the recruitment of trial participants, since we expect that patients with inguinal hernias do not always ’feel like it’ to participate in a study monitoring fertility parameters. Furthermore, only 20% of patients have bilateral hernias and male patients ≥ 45 years are often sterilized. We aim to maximize participant recruitment and retention by a detailed ‘informed consent’ and information folders.

## Current status

This study has been approved by the regional Medical Ethics Committee (VCMO, Nieuwegein, the Netherlands) and the local Ethics Board of the Diakonessenhuis Utrecht/Zeist, the Netherlands. This study is performed in accordance with the ethical standards of the Declaration of Helsinki. Recruitment of patients started on 15 March 2010. To date (August 15th, 2011) 21 patients have been included in the study. Two patients were lost to follow-up (9.5%). Depending on the number of patients needed to be included in the trial (see *Sample size considerations*), recruitment of the 76th patient is currently expected in August 2014. Analysis and reporting of data is subsequently expected 6 months later to be complete (March 2015). The MAIN study is registered in the Dutch Trial Register (NTR2208).

## Declaration of competing interests and financial disclosure

All authors hereby confirm that a Research Grant has been assigned to the Diakonnessenhuis Utrecht/Zeist, or more specifically to the Hernia Centre Zeist, by Johnson & Johnson. The Research Grant is intended to support all (completed) manuscripts on the results and complications of the Totally Extraperitoneal (TEP) endoscopic hernia repair. This study itself is not directly subject of the above mentioned Research Grant or any other financial sponsorship. Objectivity of data is therefore guaranteed and there is no conflict of interest.

There are no (other) commercial associations that might pose a conflict of interest in connection with the submitted article.

## Authors’ contributions

NSc provided the conception and design of the article. NSm and JB are responsible for the design of the initial protocol. SE is involved in the methodological design and statistical analysis. CW, LSM and RS are all responsible for analysis, writing and editing of the manuscript. All authors read and gave final approval of the version to be published.

## Financial disclosure

A Research Grant has been assigned to the Diakonessenhuis/Hernia Centre Zeist by Johnson & Johnson to (partially) support all research regarding the Totally Extraperitoneal Endoscopic hernia repair

## Pre-publication history

The pre-publication history for this paper can be accessed here:

http://www.biomedcentral.com/1471-2482/12/7/prepub

## Supplementary Material

Additional file 1 **Addendum A**: **Scrotal Ultrasound.** Click here for file
