# Supplementary material for: Male infertility after endoscopic Totally Extraperitoneal (Tep) hernia repair (Main): rationale and design of a prospective observational cohort study
Source: BMC Surg. 2012 May 21;12:7. doi: 10.1186/1471-2482-12-7 (PMC3414734; doi:10.1186/1471-2482-12-7)
Supplement: Additional file 1 — Addendum A: Scrotal Ultrasound. [file 1471-2482-12-7-S1.doc]

**SUPPLEMENTAL DATA**

**Addendum A:** Scrotal Ultrasound

**Testicular volume:**

**__________** ___________________ ml

**Thrombus plexus pampiniformis:**

- Yes
- No

**Testicular perfusion:**

| **a. testicularis** | Peak systolic  velocity (cm/s) | End diastolic  velocity (cm/s) | Resistance index | Pulsatility  index | Acceleration time |
| --- | --- | --- | --- | --- | --- |
| Inguinal canal |  |  |  |  |  |
| Extratesticular – intrascrotal level |  |  |  |  |  |
|  | Peak systolic  velocity (cm/s) | End diastolic  velocity (cm/s) | Resistance index | Pulsatility  index | Acceleration time |
| **a. capsularis** |  |  |  |  |  |
|  | Peak systolic  velocity (cm/s) | End diastolic  velocity (cm/s) | Resistance index | Pulsatility  index | Acceleration time |
| **a. intratesticularis** |  |  |  |  |  |
|  | | | | | |
